# Discordant circulating tumor DNA results favor multiple primary malignancies in patient with synchronous Merkel cell carcinoma tumors

**DOI:** 10.1016/j.jdcr.2025.12.008

**Published:** 2025-12-13

**Authors:** Nathan Kattapuram, Anthony Camargo, Kyle Murchison, Karam Khaddour, Danielle N. Margalit, Ann W. Silk, Manisha Thakuria

**Affiliations:** a Department of Dermatology, Brigham and Women’s Hospital, Boston, Massachusetts; b Department of Medical Oncology, Dana-Farber Cancer Institute, Boston, Massachusetts; c Harvard Medical School, Boson, Massachusetts; d Department of Radiation Oncology, Dana-Farber Cancer Institute and Brigham and Women’s Hospital, Boston, Massachusetts

**Keywords:** cancer genomics, circulating tumor DNA, cutaneous oncology, disease surveillance, Merkel cell carcinoma, molecular diagnostics

## Introduction

Merkel cell carcinoma (MCC) is a rare neuroendocrine malignancy. Although locoregional disease can be treated definitively with surgery or radiation, MCC has a high recurrence rate.^1^ Circulating tumor DNA (ctDNA) assays, developed from whole-exome sequencing of tumor samples and the design of multiplex polymerase chain reaction primers to recognize tumor-specific somatic nucleotide variants (SNVs) in patient blood samples, are a validated biomarker for the detection of MCC recurrence.^2^ We present a rare case of a patient with 2 synchronous MCC tumors, retrospectively favored to be different primary malignancies given incongruous ctDNA titers after disease recurrence.

## Case report

**Fig 1:**
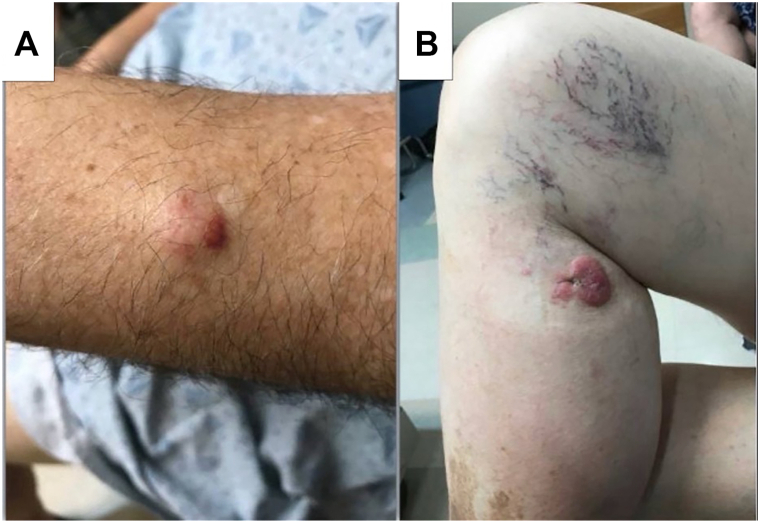
Clinical appearance of 2 concurrent extremity lesions histologically confirmed as MCC. **A,** Left upper extremity. **B,** Right lower extremity. *MCC*, Merkel cell carcinoma.

A 70-year-old male with chronic lymphocytic leukemia on observation presented with a 1-year history of a 3.7 cm pink plaque on the medial right knee (Fig 1). The lesion had grown rapidly in the previous 3 months and developed satellite metastases, confirmed histologically to be MCC. During examination, the patient was noted to have a flesh-colored, 2.1 cm nodule on the dorsum of the left forearm. A punch biopsy revealed MCC. Staging positron emission tomography/computed tomography (PET/CT) was negative for additional sites of disease.

Staging this patient was challenging. The synchronous tumors could represent either a primary tumor with an isolated distant cutaneous metastasis or 2 primary malignancies. The multidisciplinary team configured management to encompass different staging possibilities. The prospect of 2 separate primary malignancies with locoregional disease burden warranted curative-intent treatment to both sites. For the left forearm lesion, the patient underwent a wide excision with positive deep margins. One sentinel lymph node was identified in the left axilla with biopsy consistent with MCC, 0.1 mm in widest dimension, without extranodal extension. While his left arm incision was healing from surgery, the patient started definitive radiation therapy to the right lower extremity lesion. The multidisciplinary team favored definitive radiation over guideline-directed, first-line surgical management because resecting this larger tumor would have prolonged recovery time and higher risk of complication, potentially delaying initiation of postoperative radiation. The satellite metastases on the right lower extremity indicated that MCC was already seeding local dermal lymphatic channels. Hence, the multi-disciplinary team forwent a sentinel lymph node biopsy and administered prophylactic radiation to the right inguinal nodes. As distant metastatic disease could not be ruled out, the patient also started immunotherapy with pembrolizumab. A restaging PET scan, ordered given the high suspicion for metastatic disease during radiation planning for the left upper extremity, demonstrated new left epitrochlear lymphadenopathy. The patient underwent radiation therapy to the left forearm, epitrochlear nodes, and axilla. Subsequently, the patient’s scans showed no evidence of disease, and he completed 16 cycles of pembrolizumab over the course of a year.

**Fig 2:**
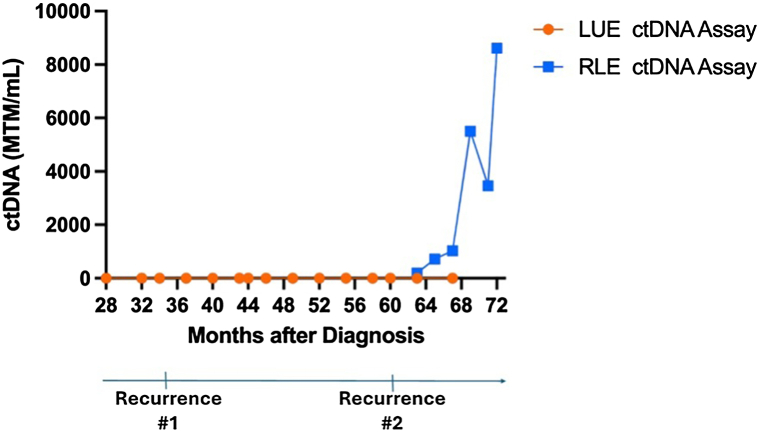
Discordant circulating tumor DNA (ctDNA) assays reflect origin of MCC recurrence. The left upper extremity (LUE) specimen from the wide local excision was used to design the primers for the first ctDNA assay, which did not become detectable with the first or second recurrence. At 63 months following diagnosis, a second ctDNA assay from the right lower extremity (RLE) tumor was conducted and detectable after the second recurrence, suggesting that the RLE tumor was a genetically distinct primary tumor responsible for the recurrences. *MCC*, Merkel cell carcinoma.

When the patient was diagnosed and initially presented to our comprehensive cancer center, ctDNA assays were not used clinically for MCC. The patient’s first ctDNA assay, developed from the tumor on the left arm, was performed 28 months after diagnosis and was undetectable (Fig 2). At this point, only 1 set of bespoke tumor-specific primers could be designed and applied per patient due to logistic constraints.

Three years after diagnosis, surveillance PET/CT highlighted a new intensely avid right inguinal node. Unexpectedly, the patient’s ctDNA remained undetectable. The right inguinal lymph node biopsy was consistent with metastatic MCC. The patient restarted immunotherapy and experienced a complete response. Following a complete response, the patient discontinued pembrolizumab after 9 infusions.

Roughly 1 and a half years after stopping his second course of pembrolizumab, he developed a recurrence in the right inguinal and pelvic lymph nodes. Again, ctDNA from the left upper extremity tumor remained undetectable. By this time, previous logistic constraints had eased such that more than 1 set of primers could be designed per patient, allowing for ctDNA assay results specific to the right lower extremity tumor. The result was elevated at 197.47 Mean tumor molecules/mL. Discrepant results between the 2 ctDNA assays suggested that the patient had 2 genomically distinct, synchronous primary malignancies on the left upper and right lower extremities, and that the disease progression was attributable to metastases from the right lower extremity tumor. The patient experienced disease progression on pembrolizumab. Despite additional lines of therapy, the patient died from his malignancy.

## Discussion

We present a case of a patient treated for synchronous MCC tumors who experienced disease recurrence in which discrepant ctDNA findings both identified the source of recurrence and indicated that the synchronous MCC tumors likely represented unrelated primary malignancies. Differentiating between multiple primary MCC tumors and distant cutaneous metastases has significant implications for staging, management, and surveillance. Prior reports have used array-based comparative genomic hybridization and next-generation sequencing to confirm clonal divergence in cases of suspected multiple primary MCC tumors.^3^^,^^4^ In the ctDNA assay used here, multiplex polymerase chain reaction primers are designed to target up to 16 tumor-specific SNVs in patient blood samples. If at least 2 SNVs are detected, a positive ctDNA result is issued.^2^ The ctDNA assay derived from the left upper extremity tumor was undetectable at the time of the second recurrence whereas the assay from the right lower extremity tumor was positive. The discordant results highlight minimal overlap in selected SNVs between the synchronous tumors. While this minimal overlap likely reflects the identities of the synchronous tumors as separate primary malignancies, it is possible but unlikely that one of the synchronous tumors was a distant cutaneous metastasis with substantial clonal drift from the primary malignancy. We cannot rule out the latter because we do not have access to the sequencing data from the vendor, but the high positive predictive value of this ctDNA assay for recurrence, including distant metastases, suggests that the typical extent of clonal drift between primary and metastatic MCC is not enough to cause false negative results.^2^ Therefore, the parallel use of ctDNA assays derived from different sites during surveillance for a patient, without evidence of disease after initial treatment of synchronous tumors, can localize the site responsible for a recurrence and credibly inform suspicion for multiple primary malignancies in retrospect.

## Conflicts of interest

Dr Silk reports receiving grants/research support (to the Institution) from CheckPoint Therapeutics, Marengo, Merck, Natera, and Regeneron; consulting fees from Regeneron and Sun Pharma; royalties from UpToDate, Inc, and stock ownership in Illumina, Inc, and Grail, Inc. Dr Thakuria served on the Incyte Advisory Board in May of 2023. Authors Kattapuram, Camargo, Murchison, and Drs Khaddour and Margalit have no conflicts of interest to declare.
